# Serum soluble toll-like receptor 4 and risk for clinical severity in COVID-19 patients

**DOI:** 10.1186/s41479-023-00121-9

**Published:** 2024-01-05

**Authors:** Maha E. Houssen, Marwa O. Elmaria, Dina Badr, Rasha El-Mahdy, Mayada A. Ghannam, Shaimaa El-Ashwah, May Denewer, Metwaly Ibrahim Mortada

**Affiliations:** 1 Biochemistry Department Faculty of Pharmacy Damanhour University, Damanhour, 22511 Egypt; 2 Chest Medicine Department Faculty of Medicine, Mansoura University, Mansoura, 35516 Egypt; 3 Department of Medical Microbiology and Immunology Faculty of Medicine, Mansoura University, Mansoura, 35516 Egypt; 4 Hematology Unit, Clinical Pathology Department Faculty of Medicine, Mansoura University, Mansoura, 35516 Egypt; 5 Clinical Hematology, Department of Internal Medicine, Oncology Centre, Faculty of Medicine, Mansoura University, Mansoura, 35516 Egypt

**Keywords:** Covid19, Toll like receptor, Soluble toll like receptor 4, Covid 19 complications

## Abstract

Toll-like receptor 4 (TLR4) signaling mediates sustained systemic inflammation in(COVID)-19 patients. We aimed to assess the serum levels of sTLR4 and sCD14 as negative regulators of Toll like receptor signaling and their association with laboratory markers and clinical severity in covid 19 patients. Ninety-eight patients with COVID-19 (70 severe and 28 non-severe) were enrolled in the study. Serum sCD14 andsTLR4were determined by ELISA. A significant increase in serum sTLR4 and sCD14 levels was detected in severe compared to non severe COVID19 patients.Receiver operating characteristic curve (ROC) analysis revealed significant diagnostic potential of serum sTLR4 and sCD14 in covid19 patients.We conclude that Serum sTLR4 and sCD14 may be promising clinical severity markers for COVID19 patients.

## Introduction

The coronavirus disease (COVID)-19 epidemic outbreak culminated in a global health disaster in 2020 [1]. The clinical symptoms of patients with COVID-19 range from mild (fever, cough, myalgia, sputum production, headache, hemoptysis, diarrhea and dyspnea) to severe lung inflammation, severe pneumonia and acute respiratory distress syndrome (ARDS**)** [2]. It can also potentially impact several organs in vulnerable people, causing cardiac and renal complications, as well as death [3]. Systemic inflammation in patients with COVID-19 is mediated by toll-like receptors (TLRs) and nod-like receptors, which are pattern recognition receptors expressed in a range of innate immune cells found in the alveolar milieu, including monocytes, macrophages and dendritic cells [4, 5]. TLR4 is an innate immune receptor which has the greatest affinity and protein interaction with the severe acute respiratory syndrome coronavirus 2 (SARS-CoV-2) spike glycoprotein, which promotes,excessive immune response, systemic inflammation, myocarditis,ARDS and damage to numerous organs in covid19 patients [6, 7]. TLR4 signaling is regulated dynamically to avoid chronic systemic inflammation and tissue damage. A variety of endogenous mechanisms negatively regulate TLR4 signaling, including cell membrane-bound TLR suppressors and soluble TLRs (sTLRs) [8]. Extracellular sTLRs function as decoy receptors, preventing ligand-induced signaling [9]. Soluble TLR4 (sTLR4) as decoy receptor and soluble CD14 (sCD14) as coreceptor for TLR4 receptor have been found to inhibit lipopolysaccharide (LPS)-induced nuclear factor-κB activation and TNF generation, [10, 11]. STLR4 inhibits TLR4-mediated signaling, possibly by interfering with receptor–ligand associations [10], While soluble CD14 is involved in regulating TLR4-induced cytokine release, via direct binding to LPS and altering the signaling patterns [12–16]. Collectively, we postulate that inhibiting TLR4 by endogenous negative regulators sTLR4 and sCD14 may improve patient outcomes by preventing systemic infection and dampening the inflammatory response in patients withCOVID-19. On this basis, the aim of the present study was to assess the serum levels of sTLR4 and sCD14 in patients with COVID-19,to examine their relationship with hematological abnormalities and to investigate their predictive value as a clinical severity marker for patients with COVID-19.

## Patients and methods

### Patients

In this cross-sectional study, 98 patients with nasopharyngeal swab reverse transcription-quantitative (RT-q) PCR-confirmed SARS-CoV-2 infection and without associated chronic disease were admitted to Mansoura university hospital COVID-19 isolation treatment center. The studied patients were selected from 300 patients admitted to the hospital from march 2020 to december 2020. None of the studied patients were assigned to any therapeutic regimens for COVID-19(such as systemic corticosteroids and hydroxychloroquine) before blood samples were taken. Patients were classified into two groups: Severe COVID-19 patients (SCP group;*n* = 70)and non-severeCOVID-19 patients (NSCP group;n = 28). Each individual enrolled in this study provided informed consent. The study was authorized by the Ethical Committee of the Faculty of Medicine of Mansoura University (approval no. R.21.9.1450; Egypt).

Patients were categorized according to the World Health Organization (WHO) severity definitions as follows: ARDS, sepsis, septic shock or other disorders that would usually necessitate the administration of life-sustaining therapies, such as mechanical ventilation (invasive or non-invasive) or vasopressor therapy, severe COVID-19 is characterized as an oxygen saturation of < 90% on room air, a respiratory rate of > 30 breaths per minute in adults, or signs of significant respiratory distress in adults (such as accessory muscle use and inability to complete full sentences), as well as non-severe COVID-19, which is described as the absence of any serious or critical COVID-19 criteria(“COVID-19 Clinical Management: Living Guidance”, 2021).

### Exclusion criteria

This study excluded patients with systemic chronic illnesses (such as hypertension, diabetes, chronic liver disease, malignancy or systemic autoimmune disease).

## Methods

### Blood sampling

A total of 10 ml whole venous blood was taken from each participant via venipuncture with sterile disposable plastic syringes and divided into three aliquots. For a full blood count (CBC), the initial aliquot of 2 ml was collected into blood collection tubes containing K2EDTA. The second 2.5 ml aliquot was collected into 3.2% sodium citrate anticoagulated tubes for prothrombin, international normalized ratio (INR) and D-dimer measurement. The third aliquot was collected in plain dry tubes and allowed to clot at room temperature for 30 min before being centrifuged at 4,000 rpm for 10 min to separate serum. The separated serum was divided into two aliquots: One was used for biochemical examinations of serum alanine aminotransferase (ALT) and aspartate transaminase (AST) activities, serum creatinine levels, serum ferritin, serumC-reactive protein (CRP) and serum lactate dehydrogenase (LDH), and the other was stored at -20˚C for determination of serum sTLR4 and sCD14 levels.

### RT-qPCR detection of COVID-19

Polyester flocked swabs were used to collect throat and nasopharyngeal swabs from all suspected SARS-CoV-2-infected patients. SARS-CoV-2 RNA was detected using a fully automated QIAGEN QIAcube system (Qiagen, Inc.) via RT-qPCR.

### Biochemical determination of serum sCD14 by ELISA

Serum sCD14 was detected by using a Human ELISA kit (cat. no. 201–12-0318) provided by Shanghai Sunred Biological Technology Co., Ltd.

### Biochemical determination of serum sTLR4 by ELISA

Serum sTLR4 was assessed by using a Human ELISA kit (cat. no. 201–12-6874) provided by Shanghai Sunred Biological Technology Co., Ltd.

### Statistical analysis

All statistical analyses were performed using SPSS for windows version 20.0 (IBM Corp.). Variables with continuous data were explored for normality of distribution using Kolmogorov–Smirnov test. The age variable was normally distributed and was expressed as the mean ± standard deviation (SD), while the other variables showed abnormal distribution of data and were expressed as the median and interquartile range (IQR). Categorical data were expressed as number and percentage. A Student’s t-test was used for comparisons between two groups of continuous data with normal distribution, while a Mann–Whitney U test was used for comparisons between two groups of abnormally distributed data. A Chi-square test was used for comparisons between groups of categorical data. A Pearson’s correlation test was used to determine the correlations between sCD14 and sTLR4 with other variables with continuous data. Receiver operating characteristic (ROC) curve analysis was performed to determine the ability of the serum sCD14 and sTLR4 levels to discriminate SCPs from NSCPs and the area under the curve (AUC) was determined. Binary regression analysis for factors predicting the severity of COVID-19 was also performed. *P* < 0.05 was considered to indicate a statistically significant difference.

### Sample size

Based on data from literature (Rialet al., 2020) [17], considering level of significance of 5%, and power of study of 80%, the sample size was calculated using the following formula: Sample size = [(Z_1-α/2_)^2^.SD^2^]/d^2^, where, Z_1-α/2_ at 5% type 1 error (*p* < 0.05) is 1.96, SD = standard deviation of variable and d = absolute error or precision. So, Sample size = [(1.96)^2^.(1267.1)^2^]/(250.9)^2^ = 97.9. Based on the above formula, the sample size required for the study is 98.

## Results

### Demographics of patients with COVID-19

A total of 28 patients with COVID-19 were classified into the NSCP group, while 70 patients were classified into the SCP group. When comparing SCPs and NSCPs, a substantial drop in oxygen saturation was found (*P* = 0.001).SCPs had a significantly worse survival rate (*P* = 0.004) than NSCPs. SCPs had a higher radiological severity grade than NSCPs (*P* = 0.017). Dyspnea was significantly worse in the SCP group compared with the NSCP group (*P* = 0.044) (Table 1).

**Table 1:** Demographic data of covid 19 patients

	NSCP group(*n* = 28)	SCP group (*n* = 70)	
	Median [IQR]	Median [IQR]	P
**Age (Years) (n, %)** (Mean ± SD)	59.4 ± 15.0	63.9 ± 13.2	0.143
**Sex (n, %)**			
Male	12, 42.9%	27, 38.6%	
Female	16, 57.1%	43, 61.4%	0.695
**Symptoms**			
Dyspnea	14, 50.0%	50, 71.4%	0.044
Dry Cough	14, 50.0%	36, 51.4%	0.898
Productive Cough	10, 35.7%	19, 27.1%	0.401
Fatigue	14, 50.0%	34, 48.6%	0.898
Bone Aches	10, 35.7%	27, 38.6%	0.792
Fever	18, 64.3%	38, 54.3%	0.366
Diarrhea	6, 21.4%	13, 18.6%	0.747
Anorexia	4, 14.3%	9, 12.9%	0.851
Taste Loss	7, 10.0%	6, 21.4%	0.132
Smell Loss	6, 8.6%	4, 14.3%	0.399
Vomit	8, 28.6%	14, 20.0%	0.358
Sore Throat	0, 0.0%	5, 7.1%	0.147
Chest Pain	2, 7.1%	5, 7.1%	1.000
Nausea	0, 0.0%	2, 2.9%	0.366
O_2_ Saturation (n, %)			
< 87%	0, 0.0%	58, 82.9%	
> 87%	28, 100.0%	12, 17.1%	< 0.001
**Radiological CO-RAD Category** (n, %)			
CO-RADs 4	10, 35.7%	10, 14.3%	
CO-RADs 5	18, 64.3%	60, 85.7%	0.017
**Survival** (n, %)			
Dead	4, 14.3%	32, 45.7%	
Survived	24, 85.7%	38, 54.3%	0.004

*NSCP* non severe covid patients, *SCP* Severe covid patients

### Hematological and biochemical laboratory abnormalities in patientswithCOVID-19

The hemoglobin (Hb) levels in the SCP group were lower than in the NSCP group(*P* = 0.028). White blood cells (WBCs), neutrophils, CRP, ferritin and D-dimer were all considerably lower in the NSCP group than in the SCP (*p* < 0.001). However, it was found that the lymphocyte count in NSCPs was higher than in SCPs (*P* = 0.001). Between the NSCP and SCP groups, there were no significant variations in serum creatinine, serum alanine transaminase (ALT) and serum aspartate transaminase (SGOT)activities, serum albumin, plasma INR and platelets count (Table 2).

**Table 2:** Hematological parameters of COVID-19 patients groups

	NSCP group(*n* = 28)	SCP group (*n* = 70)	
	Median [IQR]	Median [IQR]	P
**Complete blood picture ( CBC)**			
Hb (g/dl)	12.1 [3.10]	11.25 [2.93]	0.028
WBCs count (cells/mm^3^)	5.5 [9.71]	8.3 [5.68]	0.031
Neutrophils%	48.6 [45.0]	79.6 [17.5]	< 0.001
Lymphocytes%	37.0 [45.0]	13.5 [13.7]	< 0.001
Platelet count (cells/mm^3^)	206.5 [68.0]	207.0 [117.4]	0.863
**Serum Parameters**			
AST (U/L)	30.0 [34.0]	31.5 [29.0]	0.514
ALT (U/L)	26.0 [20.0]	26.5 [27.0]	0.184
LDH (U/Ll)	242.5 [237.0]	440.0 [324.8]	< 0.001
Serum Ferritin (μg/L)	399.0 [103.0]	546.0 [262.0]	< 0.001
D-dimer (μg/ml)	0.2 [0.0]	0.5 [0.5]	< 0.001
CRP	77.0 [78.8]	98.5 [133.5]	0.021
INR	1.1 [0.2]	1.2 [0.3]	0.315
Creatinine (mg/dl)	1.1 [0.5]	1.0 [0.6]	0.900
Albumin (gm/dl)	3.4 [0.4]	3.2 [0.6]	0.533

*Hb* Hemoglobin, *WBCS* White blood cells, *ALT* Alanine transaminase, *LDH* lactate dehydrogenase, *CRP* C-reactive protein, *STLR4* soluble toll like receptor 4, *INR* international normalized ratio, *AST* Aspartate transaminase, *NSCP* non severe covid patients, *SCP* Severe covid patients

### Serum sTLR4 and serum sCD14 in patientswithCOVID-19

**Fig. 1:**
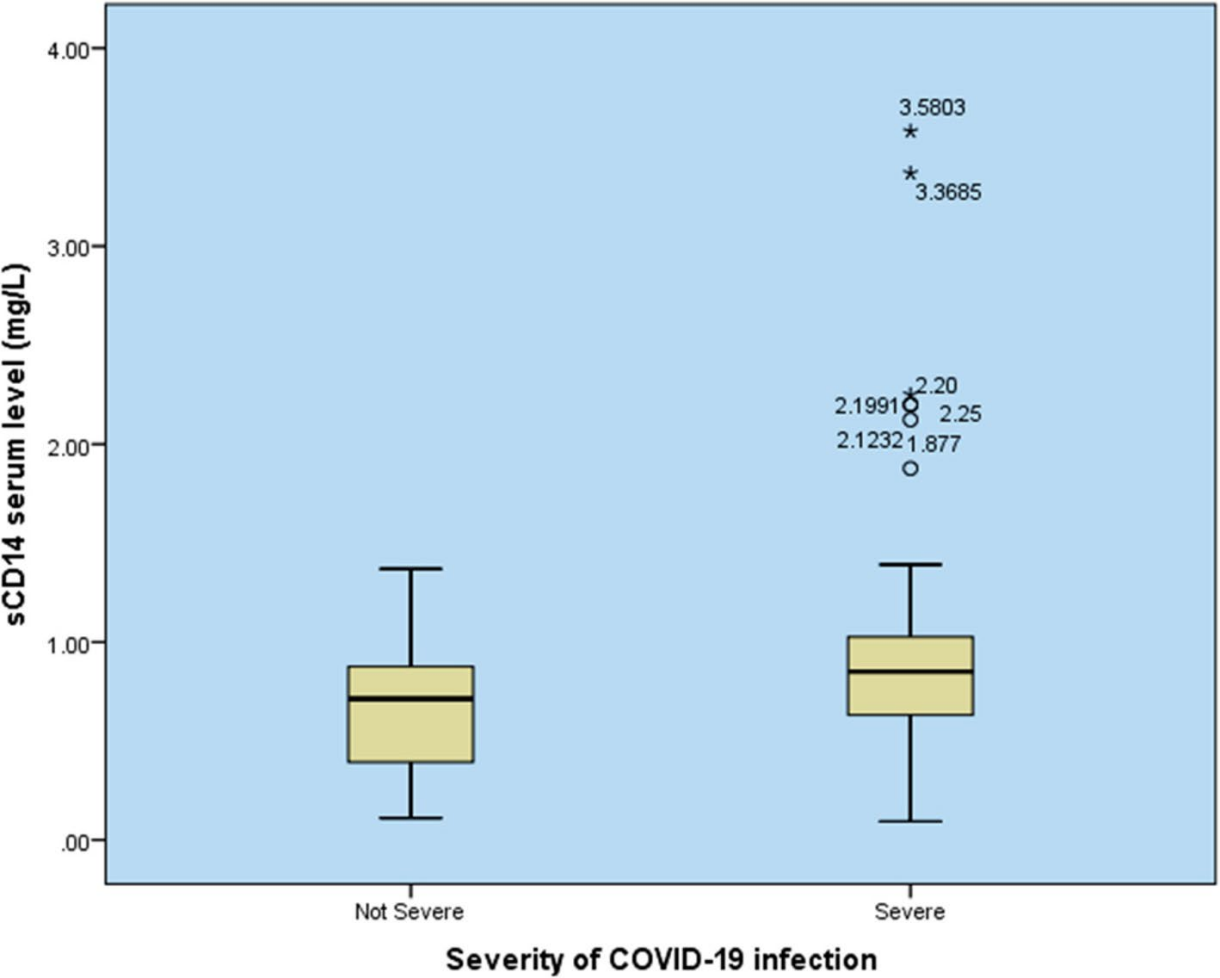
Comparison of serum sCD14 levels between severe and non-severe COVID-19 patients

**Fig. 2:**
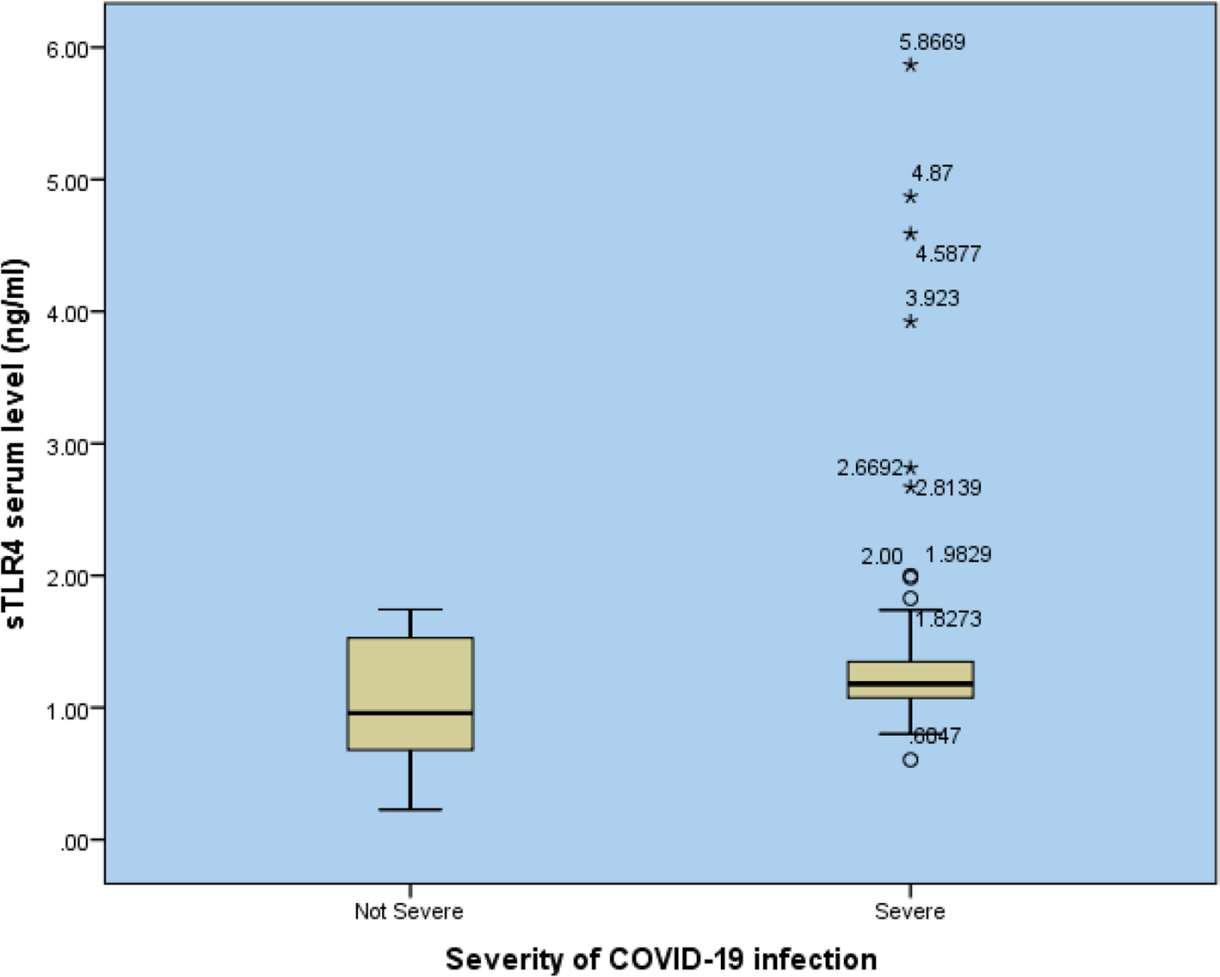
Comparison of serum sTLR4 levels between severe and non-severe COVID-19 patients STLR4: soluble toll like receptor 4

The SCP group showed a significant increase in both serum sTLR4 and sCD14 levels compared with the NSCP group (*P* = 0.01 and 0.017), respectively (Table 3 and Figs. 1 and 2).

**Table 3:** Serum sTLR4 and sCD14 levels in patients with COVID-19

	NSCP group(*n* = 28)	SCP group (*n* = 70)	
	Median [IQR]	Median [IQR]	P
**Immunological Biomarkers**			
sCD14 (mg/L)	0.71 [0.52]	0.85 [0.41]	0.011
sTLR4 (ng/ml)	0.96 [0.87]	1.18 [0.28]	0.017

*STLR4* soluble toll like receptor 4, *NSCP* non severe covid patients, *SCP* Severe covid patients

### Regression analysis

Binary regression analysis was performed to assess the risk of COVID-19 severity for demographic factors, hematological abnormalities, serum sTLR4 and serum sCD14 levels (Table 4). COVID-19 patients with a lower lymphocyte count were more likely to develop severe symptoms (*P* = 0.006) OR (4.02). Moreover, patients with COVID-19 who presented with lower O_2_ saturation ratios were at high risk for developing more severe symptoms (*P* < 0.001), OR(8.19). In addition, patients with higher circulating levels of sTLR4 (*P* = 0.010),OR(3.36) and sCD14 (*P* = 0.012), OR(3.38) were much more vulnerable to severe symptoms of the disease. (Table 4).

**Table 4:** Linear regression analysis for factors predicting the severity of COVID-19

	Unstandardized Coefficients		Standardized		
	B	Std. Error	Coefficients Beta	t	p
(Constant)	1.094	0.353		3.101	0.003
Hb (g/dl)	0.013	0.009	0.146	1.447	0.151
WBCs count (cells/mm^3^)	0.090	0.054	0.167	1.658	0.101
Neutrophils %	-0.002	0.003	-0.086	-0.604	0.547
Lymphocytes %	-0.009	0.003	-0.387	-2.835	0.006
LDH(U/L)	-0.068	0.055	-0.125	-1.230	0.222
Serum Ferritin (μg/L)	0.001	0.001	0.079	1.306	0.195
D-dimer (μg/ml)	0.025	0.055	0.029	0.465	0.643
CRP	0.001	0.000	0.094	1.638	0.105
O_2_Saturation	-0.480	0.065	-0.522	-7.336	< 0.001
CO-RAD category	0.136	0.081	0.170	1.683	0.096
sCD14(mg/L)	0.181	0.069	0.161	2.620	0.010
sTLR4(ng/ml)	0.119	0.054	0.221	2.552	0.012

*Hb* Hemoglobin, *WBCS* White blood cells, *LDH* lactate dehydrogenase, *CRP* C-reactive protein, *STLR4* soluble toll like receptor 4, *NSCP* non severe covid patients, *SCP* Severe covid patients

### Diagnostic performance of serum sTLR4 and sCD14

**Fig. 3:**
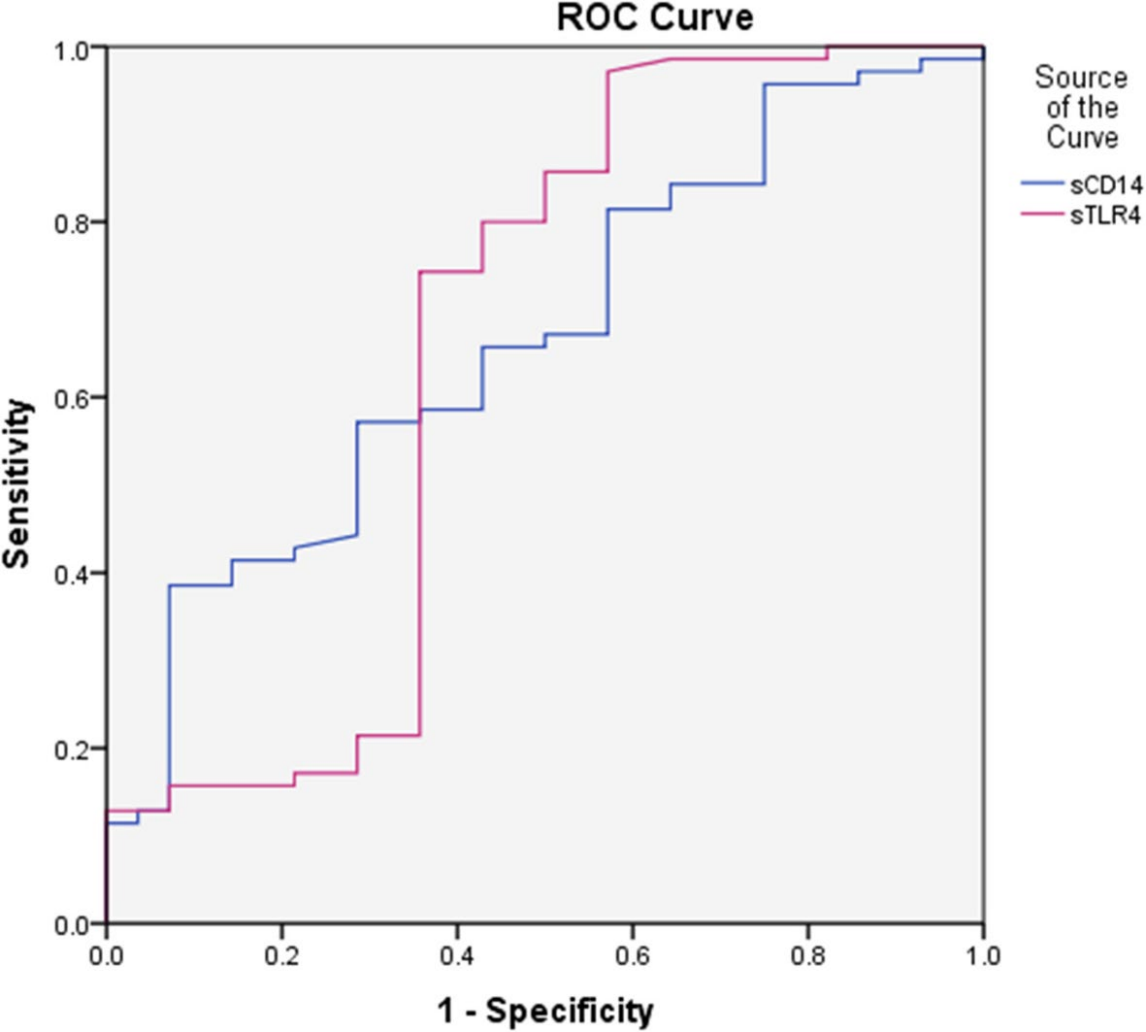
ROC curve analysis for ability of serum sCD14 and sTLR4 for discrimination between patients with non severe and severe COVID-19 infection (AUC for sCD14 = 0.665 and for sTLR4 = 0.655)

The ROC curve analysis was employed to assess the prediction value of sTLR4 and sCD14 as markers of COVID-19 severity, which revealed moderate diagnostic performance as measured by the AUC for sTLR4 (AUC = 0.655,CI 95%) with sensitivity 27.16% and specificity 68.97% and sCD14 (AUC = 0.665,CI 95%) with sensitivity74.07% and specificity 50.00% (Fig. 3) (Tables 5, and 6).

**Table 5:** Correlation of serum sCD14 and sTLR4 with age and laboratory parameters of patient swith COVID-19

	sCD14		sTLR4	
	r	P	R	P
Age (years)	0.167	0.101	0.185	0.068
Hb concentration (g/dl)	-0.122	0.232	-0.055	0.589
WBCs (cells/mm^3^)	0.287^**^	0.004	0.287^**^	0.004
Neutrophils %	0.236^*^	0.019	0.203^*^	0.045
Lymphocytes %	-0.243^*^	0.016	-0.222^*^	0.028
Platelet count	-0.104	0.310	-0.074	0.471
AST(U/L)	-0.235-^*^	0.020	-0.206^*^	0.042
ALT(U/L)	-0.238-^*^	0.018	-0.111	0.278
LDH (U/L)	0.039	0.701	0.104	0.306
Serum Ferritin (μg/L)	0.083	0.415	0.094	0.355
D-dimer(μg/ml)	0.094	0.357	0.012	0.904
CRP	0.044	0.670	0.084	0.410
INR	-0.102	0.317	-0.137	0.180
Creatinine (mg/dl)	-0.070	0.493	-0.044	0.666
Albumin(gm/dl)	-0.054	0.599	-0.054	0.598
sCD14(mg/L)			0.823^**^	< 0.001
sTLR4(ng/ml)	0.823^**^	< 0.001		

*Hb* Hemoglobin, *WBCS* White blood cells, *AST* Aspartate transaminase, *ALT* Alanine transaminase, *LDH* lactate dehydrogenase, *CRP* C-reactive protein, *STLR4* soluble toll like receptor 4, *INR* international normalized ratio

**stong positive correlation, *positive correlation

**Table 6:** Diagnostic value of the sTLR for differentiation between severe and non-severe COVID – 19 at a cut point of at a cut point of 1.305 pg/ml

	Value	95% CI
Sensitivity	27.16%	17.87% to 38.19%
Specificity	68.97%	55.46% to 80.46%
Disease prevalence	58.27%	49.61% to 66.57%
Positive Predictive Value	55.00%	41.99% to 67.36%
Negative Predictive Value	40.40%	35.28% to 45.74%
Accuracy	44.60%	36.18% to 53.27%

### Relationships betweensTLR4 and sCD14 with laboratory parameters

**Fig. 4:**
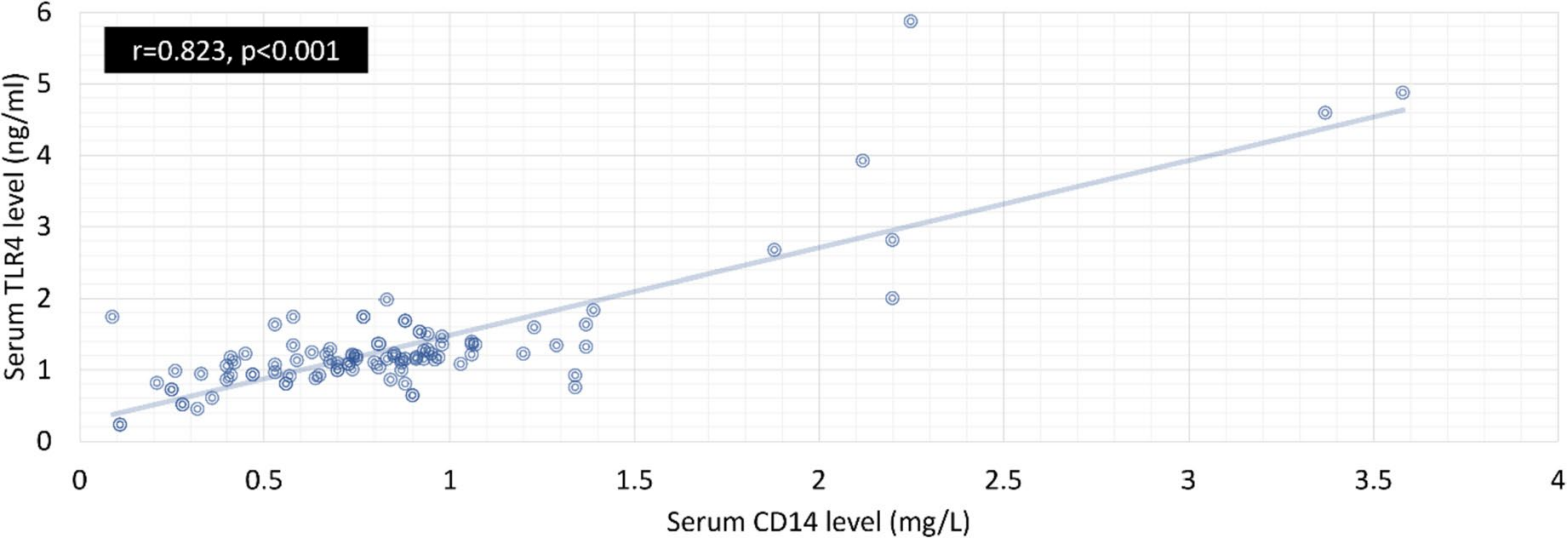
Correlation between Serum CD14 and sTLR4 levels in covid19 patients

Serum sTLR4 showed a strong positive correlation with neutrophil count (*P* = 0.045), WBC count (*P* = 0.004), lymphocyte count (*P* = 0.028), AST activity (*P* = 0.042) and serum sCD14 levels (*P* < 0.001, *r* = 0.823) (Table 7 and Fig. 4). Concerning serum sCD14, there was a substantial positive correlation between its serum levels and WBC count (*P* = 0.004), neutrophil count (*P* = 0.019), lymphocyte count (*P* = 0.016), AST activity (*P* = 0.020), ALT activity (*P* = 0.018) and serum sTLR4 (*P* < 0.001, *r* = 0.823) (Table 7 and Fig. 4).

**Table 7:** Diagnostic value of the CD14 for differentiation between severe and non-severe COVID – 19 at a cut point of at a cut point of 0.67 pg/ml

	Value	95% CI
Sensitivity	74.07%	63.14% to 83.18%
Specificity	50.00%	36.58% to 63.42%
Disease prevalence	58.27%	49.61% to 66.57%
Positive Predictive Value	67.42%	60.81% to 73.40%
Negative Predictive Value	58.00%	46.84% to 68.39%
Accuracy	64.03%	55.46% to 71.99%

## Discussion

TLRs are a type of pattern recognition receptor that play an important function in the immune system of the host [18]. They play a key role in systemic inflammation by activating the MAPK/NFB signaling pathway, which results in cytokines production and the maintenance of chronic inflammation [19]. To counteract systemic inflammation caused by TLRS activation, sTLRs are released into the circulation from tissues and blood cells [20]. TLR4 in its soluble form suppresses TLR4-mediated signaling as well,it likely mediates this effect by interfering with receptor-ligand interactions [21]. sCD14, as a co receptor of TLR4 signaling on the other hand, is involved in controlling TLR4-induced cytokine release through directly binding LPS to alter TLRS signaling patterns [22].

Thus, the goal of the present study was to gain insight into the serum levels of sTLR4 and sCD14 in SCPs and NSCPs to assess their correlation with hematological abnormalities and to investigate their potential clinical utility as a clinical severity marker forCOVID-19 patients.To the best of our knowledge, this is the first study that evaluated the serum levels of sTLR4 as an endogenous negative regulator of TLR4 signaling in patients with COVID-19.

A number of hematological abnormalities were found in SCPs compared with NSCPs in the present study, including lymphopenia and low hemoglobin levels, hyperferritinemia, elevated serum LDH levels and raised circulating CRP levels. Furthermore, there was strong evidence of a specific coagulopathy risk in SCPs, as seen by the increased plasma D-dimer levels and a higher risk of thrombotic events.

The precise method by which COVID-19 lymphopenia develops is unknown, however it could be connected to lymphocyte expression of the main SARS-CoV-2 entry receptor, ACE2, and subsequent lymphoid death as a result of infection [23, 24]. This process may be aided by lymphocyte trafficking from the peripheral circulation to the lungs or other infection sites [25]. The neutrophilia reported in severe COVID-19 instances, on the other hand, can be related to a response to the cytokine storm, which has been linked to the disease's most severe symptoms [26]. Additionally, large increases were seen in the acute-phase markers ferritin and CRP. Numerous studies have found that biomarkers such as ferritin and CRP are positively correlated with elevated pro-inflammatory cytokines and are linked to COVID-19 severity and mortality [27, 28]. The increased risk of coagulopathy in SCPs was also detected in the current study, as reflected by higher plasma D-dimer levels compared with NSCPs. This finding was consistent with prior studies that indicated that a rise in D-dimer levels is linked to the development of severe disease and in-hospital mortality in patients withCOVID-19 [25]. Various factors contribute to the enhanced coagulopathy seen in severe patients, including direct endothelial injury from SARS-CoV-2 or immune cells [25], cytokine-induced coagulation cascade activation [29] and an increase in acute-phase pro-coagulants, including factor VIII and fibrinogen [25].

Lower hemoglobin levels were associated with hyperferritinemia and elevated LDH levels in SCPs which are major predictors of mortality [30, 31]. Activated innate immune response, which restricts iron availability during infections is the susceptible mechanism that can also lead to anemia, which in turn reduces oxygen delivery to the tissue and may thus play an important role in the development of multi-organ failure. On the other hand, increased ferritin levelsas acute phase protein in SCPs could be attributed to a robust inflammatory response [32, 33]. In addition, the higher levels of LDH in the examined SCPs could be attributed to severe infections that may result in tissue damageand cells apoptosis mediated by cytokines and the release of LDH.As LDH (isozyme 3) is found in lung tissue, patients with severe COVID-19 infections may discharge more LDH into the bloodstream, as a severe form of interstitial pneumonia [34].

The most important novel finding in the present study was the significantly elevated levels of serum sTLR4 and sCD14 inthe SCP group compared with the NSCP group. This finding was in line with previous research, which found that sTLRs are released into the circulation from tissues and blood cells, and their levels rise during infections and inflammatory diseases [35, 36]. TenOever*et al* [36] reported rapid elevation of sTLR4 in plasma after LPS treatment and showed that this rapid increase is a feedback mechanism to counteract TLR4 signaling activation. Furthermore, STLR4 is proposed to create a complex with myeloid differentiation factor 2(MD-2)and prevent the TLR4-MD-2 complex from forming, which is required for ligand binding and thus inhibits TLR signaling [36]. In addition, sTLR4 may interfere with LPS/TLR4 signaling by interacting with CD14 and/or LPS binding protein, both of which are required for ligand binding with TLR4 [10]. Moreover, the current study found a substantial positive association between sTLR4 and sCD14,leukocytosis, lymphopenia and neutrophilia, all of which are strong indicators of COVID-19 severity. This positive correlation between sCD14, sTLR4 and both lymphopenia andneutophilia may be attributed to the direct expression of TLRs on the surface of these cells and the impact of these receptor signaling on controlling their effector functions [5].

Furthermore, ROC analysis revealed that sTLR4 was a predictive marker for identifying SCPs and NSCPs. All of these data supported the concept that sTLR4 plays a compensatory role in reducing systemic inflammation in highly infected COVID-19 patients. The current study also found that SCPs had higher median blood levels of serum sCD14 than NSCPs, which was consistent with a recent study that detected higher serum sCD14 levels in patients with COVID-19 admitted to hospital (37).The elevated levels of sCD14 can be attributed to the compensatory role of sCD14 as a negative regulator of TLR signaling [37–39].

## Conclusion

Finally, it can be concluded that sTLR4and sCD14, as endogenous negative regulators of TLR4 signaling, may be disease severity markers with moderate sensitivity for patients with COVID-19. Future research is needed to validate these results on large patients’ cohort and to confirm the predictive significance of these regulators as a promising target for immunotherapy in patients with COVID-19 infection.

### Study limitations

Despite this study provides preliminary evidence that sTLR4 and sCD14 may serve as markers of COVID-19 severity. Some limitations should be presented as follow

- Single timepoint measurement of sTLR4/sCD14 limits ability to assess relationship with disease progression.- The patient population lacks diversity—conducted at a single center in Egypt. Results may not generalize to other populations. In other words, Small sample size from a single center limits generalizability widely.
- Lack of longitudinal follow-up data. No validation cohort to confirm findings.- No healthy control group for comparison of baseline TLR levels.- Potential confounding factors are not fully addressed in the analysis, like age and co-morbidities.- Underlying conditions that could affect TLR levels are excluded but these are important comorbidities for COVID-19.- It would be better if the cases number were more than 150 cases.—Specifics of patient treatment regimens are not reported.- Mechanistic/functional relationship between TLR levels and COVID-19 severity not fully established. Complementary functional studies are needed to delineate the mechanistic and causal relationships between sTLR4/sCD14 levels and COVID-19 disease severity that are lacking in this observational clinical study. This will better establish these markers as prognostic biomarkers and therapeutic targets.

## Data Availability

The authors will not share their data as these data confined to the participants in this research only The data that support the findings of this study are available from the corresponding author upon reasonable request.

## Abbreviations

sTLR4: Soluble toll like receptor 4
sCD14: Soluble cd14
TLR4: Toll like receptor 4
